# Effect of Hachimijiogan against Renal Dysfunction and Involvement of Hypoxia-Inducible Factor-1**α** in the Remnant Kidney Model

**DOI:** 10.1155/2011/348686

**Published:** 2011-02-24

**Authors:** Hiroshi Oka, Hirozo Goto, Keiichi Koizumi, Shin Nakamura, Koichi Tsuneyama, Yue Zhou, Michiko Jo, Takako Fujimoto, Hiroaki Sakurai, Naotoshi Shibahara, Ikuo Saiki, Yutaka Shimada

**Affiliations:** ^1^Department of Japanese Oriental Medicine, Graduate school of Medicine and Pharmaceutical Sciences, University of Toyama, 2630 Sugitani, Toyama 930-0194, Japan; ^2^Division of Pathogenic Biochemistry, Institute of Natural Medicine, University of Toyama, 2630 Sugitani, Toyama 930-0194, Japan; ^3^Department of Kampo Diagnostics, Institute of Natural Medicine, University of Toyama, 2630 Sugitani, Toyama 930-0194, Japan; ^4^Biomedical Institute, NPO Primate Agora, Inuyama, Aichi 484-0002, Japan; ^5^Department of Diagnostic Pathology, Graduate School of Medicine and Pharmaceutical Sciences, University of Toyama, 2630 Sugitani, Toyama 930-0194, Japan; ^6^Faculty of Human Development, University of Toyama, 3190 Gofuku, Toyama 930-8555, Japan

## Abstract

In chronic renal failure, hypoxia of renal tissue is thought to be the common final pathway leading to end-stage renal failure. In this study the effects of hachimijiogan, a Kampo formula, were studied with respect to hypoxia-inducible factor (HIF). Using remnant kidney rats, we studied the effects of hachimijiogan on renal function in comparison with angiotensin II receptor blocker. The result showed that oral administration of hachimijiogan for seven days suppressed urinary protein excretion and urinary 8-OHdG, a marker of antioxidant activity, equally as well as oral administration of candesartan cilexetil. In contrast, the protein volume of HIF-1**α** in the renal cortex was not increased in the candesartan cilexetil group, but that in the hachimijiogan group was increased. In immunohistochemical studies as well, the expression of HIF-1**α** of the high-dose hachimijiogan group increased compared to that of the control group. Vascular endothelial growth factor and glucose transporter 1, target genes of HIF-1**α**, were also increased in the hachimijiogan group. These results suggest that hachimijiogan produces a protective effect by a mechanism different from that of candesartan cilexetil.

## 1. Introduction

In recent years, it has been clear that chronic kidney disease (CKD) is an important risk factor for cardiovascular diseases and mortality and has been the focus of considerable attention [1]. The number of dialysis patients caused by end-stage renal disease has been increasing worldwide, regardless of the advances in treatments, such as a protein- and sodium-restricted diet, medicines containing angiotensin II receptor blocker (ARB), and kidney transplants [2]. There are various causes for the early stage of CKD, but there is a common pathway in advanced CKD as represented by interstitial fibrosis, glomerular sclerosis, and nephron destruction [3]. The kidney is sensitive to oxygen supply and is prone to sustaining damage from hypoxia. In recent years, it has been reported that chronic hypoxia in the kidney is the final common pathway to end-stage renal failure [4, 5]. Thus, therapeutic approaches against hypoxic injury in CKD patients are considered important for preventing the worsening of CKD. As a biological defense mechanism against tissue hypoxia, hypoxia-inducible factor (HIF), a heterodimeric transcription factor, plays an essential role. Activation of HIF stimulates numerous downstream target genes and protects tissue from hypoxia [6]. This effect is suggested by reports, which showed that cobalt ameliorates renal injury of renal disease model rats [7], and vascular endothelial growth factor (VEGF) enhances glomerular capillary repair and accelerates resolution of experimentally induced glomerulonephritis [8].

Hachimijiogan, which we used in this study, is a Kampo formula created more than 1800 years ago. It is composed of eight crude drugs: Cinnamomi Cortex, Aconiti Japonici Tuber, Rehmanniae Radix, Corni Fructus, Dioscoreae Rhizoma, Alismatis Rhizoma, Moutan Cortex, and Hoelen. It has been clinically used to treat many symptoms, such as lumbago, pollakisuria, cold hands and feet, nephritis, and so on [9]. In basic research, it has been reported that hachimijiogan inhibits the progression of renal dysfunction in diabetic nephropathy model rats and 5/6 nephrectomized model rats. As the mechanism of its action, reduction of uremic toxins associated with antioxidant activity and positive effects on organic change of the kidney have been reported [10, 11].

However, there are no studies reporting the effect of hachimijiogan on HIF. Therefore, we examined the effect of hachimijiogan in comparison to ARB, a mainstream drug for renal disorder, on renal dysfunction from the aspect of hypoxic injury using 5/6 nephrectomized rats, in which tissue hypoxia causes progression of renal failure [12, 13].

## 2. Materials and Methods

### 2.1. Materials

The bulk extract of hachimijiogan (batch number 2060007020), which is approved for medical use in Japan, was purchased from Tsumura Co. Ltd. (Tokyo, Japan). It consists of eight herbs: 6.0 g of Rehmanniae Radix (*Rehmannia glutinosa* Liboschitz var. *purpurea* Makino), 3.0 g of Corni Fructus (*Cornus officinalis* Siebold et Zuccarini), 3.0 g of Dioscoreae Rhizoma (*Dioscorea japonica* Thunberg), 3.0 g of Alismatis Rhizoma (*Alisma orientale* Juzepczuk), 3.0 g of Hoelen (*Poria cocos* Wolf), 3.0 g of Moutan Cortex (*Paeonia suffruticosa* Andrews), 2.5 g of Cinnamomi Cortex (*Cinnamomum cassia* Blume), 1.0 g of Aconiti Tuber (*Aconitum carmichaeli* Debeaux). 

Candesartan cilexetil was obtained from Takeda Pharmaceutical Company Ltd. (Osaka, Japan).

### 2.2. Three-Dimensional HPLC Analysis of Hachimijiogan

**Figure 1:**
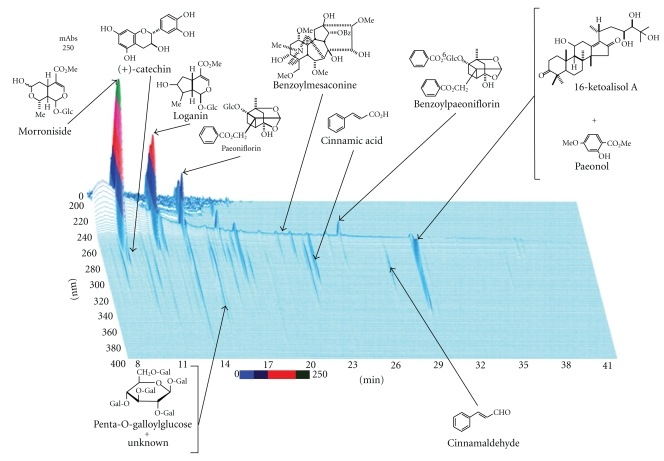
Chemical profile of hachimijiogan analyzed by three-dimensional HPLC.

For analysis of the components of hachimijiogan, aqueous extract (1 g) was extracted with 20 mL methanol under ultrasonication for 30 min. The solution was filtered through a membrane filter (0.45 *μ*m) and then subjected to high-performance liquid chromatography (HPLC) analysis using a TSK-GEL ODS-80TS column (*φ*4.6 × 250 mm, Tosoh, Tokyo, Japan) with an LC 10AD pump and an SPD-M10A absorbance detector. The elution solvents were (A) 0.05 M AcOH-AcONH_4_ and (B) CH_3_CN, and the column was eluted with a linear gradient of, by volume, 90% A and 10% B changing over 60 min to 100% B. The flow rate was 1.0 mL/min, and the effluent from the column was monitored and processed into three-dimensional data by an SPD-M10A array detector. All assigned peaks were identified by comparing their UV spectral data with those of coinjected authentic samples using the Class LC-10 Version 1.62 software package (Shimadzu, Kyoto, Japan). The three-dimensional HPLC profile of hachimijiogan extract is shown in Figure 1. The major components of hachimijiogan were morroniside, loganin, paeoniflorin, penta-O-galloyl glucose, benzoylmesaconine, benzoylpaeoniflorin, 16-ketoalisol A, paeonol, cinnamic acid, and cinnamaldehyde.

### 2.3. Animals and Treatments of Animals

All experimental procedures were performed in accordance with the standards established by the “Guide for the Care and Use of Laboratory Animals at the University of Toyama”. Fifty 6-week-old male Sprague-Dawley rats were purchased from Japan SLC Inc. (Hamamatsu, Japan) and kept in an automatically controlled room (temperature about 23°C and humidity about 60%) with a conventional dark/light cycle. The animals were kept in metabolic cages, and 24-hour urine samples were collected. Blood pressure was determined by tail cuff system (MK2000, Muromachi Kikai Co., Ltd., Tokyo, Japan) in a conscious state. At 7 weeks old, 40 rats underwent 5/6 nephrectomy under anesthesia with sodium pentobarbital (50 mg/kg body weight, i.p.) by ablation of approximately 2/3 of the left kidney, and then removal of the right kidney by ligation of renal artery, vein, and ureter 1 week later. After recovery from the operation (after 1 week), the 5/6 nephrectomized (5/6Nx) rats were randomly divided into four groups (control and three treatment groups, *n* = 10/group). One more group of rats had undergone a sham operation (*n* = 10). During the experimental period, all groups were fed a standard chow. The sham and control groups were fed water, and the other three surgical groups were fed a solution of hachimijiogan extract orally at a dose of 220 mg/kg body weight/day (low-dose hachimijiogan), 660 mg/kg body weight/day (high-dose hachimijiogan), or a solution of candesartan cilexetil orally at a dose of 3 mg/kg body weight/day, respectively, by gastric gavage. These doses of hachimijiogan for rats were approximately 3 times and 10 times the human dose of hachimijiogan. After 7 days of treatment, the rats were sacrificed, and blood samples were obtained. The kidneys were removed from each rat, frozen quickly, and kept at −80°C until analysis.

### 2.4. Analysis of Serum and Urine Samples

Serum levels of Albumin were determined by SRL, Inc. (Tokyo, Japan). Serum levels of urea nitrogen (BUN) and creatinine (s-Cre) were determined using commercial kits (BUN Kainos and CRE-EN Kainos purchased from Kainos Laboratories, Inc., Tokyo, Japan). Urinary protein (u-Pro) excretion levels were determined using commercial reagents (Micro TP-test, Wako Pure Chemical, Osaka, Japan). Creatinine clearance (Ccr) was calculated on the basis of urinary creatinine, serum creatinine, urine volume, and body weight using the following equation: Ccr (mL/(kg body weight)/min) = {urinary Cre (mg/dL) × urine volume (mL)/serum Cre/(mg/dL)} × {1,000/body weight (g)} × {1/1,440 (min)}. 8-Hydroxy-deoxyguanosine (8-OHdG) content in 24-hour urine samples was measured by ELISA kit (8-OHdG Check, JaICA, Nikken SEIL Co., Shizuoka, Japan).

### 2.5. Real-Time RT-PCR

Total RNA was prepared using the RNeasy Mini kit (QIAGEN, Valencia, CA, USA). First-strand cDNA was synthesized by SuperScript II reverse transcriptase (Invitrogen, Carlsbad, CA, USA). cDNA was amplified quantitatively using SYBR Premix Ex Taq (TaKaRa-Bio, Otsu, Japan). The primer sequences are summarized in Table 1. Real-time quantitative RT-PCR was performed using an ABI Prism 7300 sequence detection system (Applied Biosystems, Foster City, CA, USA). All data were normalized to *β*-actin mRNA.

### 2.6. Protein Preparation and Western Blotting

The cortex was dissected from the frozen kidney and homogenized in buffer A (10 mM HEPES pH 7.9, 10 mM KCl, 0.1 mM EDTA, 0.1 mM EGTA, 1 mM DTT, 1 mM PMSF, 20 mM *β*-glycerophosphate, 0.1 mM sodium orthovanadate, 10 *μ*g/mL aprotinin, and 10 *μ*g/mL leupeptin) and chilled on ice for 15 min. Next, 25 *μ*L of 10% Nonidet P-40 was added and the suspension was vigorously vortexed for 10 s and kept on ice for 5 min. The nuclear pellets were washed with 100 *μ*L of buffer A and suspended in 50 *μ*L of buffer C (20 mM HEPES pH 7.9, 0.4 M NaCl, 1 mM EDTA, 1 mM EGTA, 1 mM DTT, 1 mM PMSF, 20 mM *β*-glycerophosphate, 1 mM sodium orthovanadate, 10 *μ*g/mL aprotinin, 10 *μ*g/mL leupeptin). The mixture was kept on ice for 15 min with frequent agitation. Nuclear extracts were prepared by centrifugation at 15,000 rpm for 5 min. 

Kidney lysates were resolved by SDS-PAGE and transferred to Immobilon-P nylon membrane (Millipore, Bedford, MA, USA). The membrane was treated with BlockAce (DS pharma Co. Ltd., Suita, Japan) overnight at 4°C and probed with primary antibodies. An antibody against HIF-1*α* (H1alpha67) was purchased from Abcam (Cambridge, UK). Lamin B was used as an internal control. Antibodies against Lamin B (C-20) were purchased from Santa Cruz Biotechnologies (Santa Cruz, CA, USA). Enhancer solutions (Can Get Signal; Toyobo, Osaka, Japan) were used for the dilution. The antibodies were detected using horseradish peroxidase-conjugated antimouse and antigoat IgG (Dako Cytomation, Glostrup, Denmark) and visualized with the ECL system for Lamin B and ECL-plus for HIF-1*α* (GE Healthcare, Buckinghamshire, UK).

### 2.7. Histology and Immunohistochemistry

Rats were deeply anesthetized by an intraperitoneal injection of pentobarbital sodium (50 mg/kg body weight). Kidney was rapidly excised and immediately immersed in 4% paraformaldehyde and embedded in paraffin. Sections (5 *μ*m thick) were routinely stained with hematoxylin and eosin. Mouse monoclonal antibody against HIF-1*α* (H1alpha, 1 : 25 diluted; Novus Biologicals, Littleton, CO, USA) was used for immunohistochemical staining of kidney as previously described [14]. For detecting primary antibodies on rat tissue specimens, M.O.M. kit (Vector, Burlingame, CA, USA) was used for special blocking. Tissue sections were cut at 5 micrometers from tissue blocks and placed on slides. After deparaffinization, sections were soaked in target retrieval solution (TRS, pH 6.1, Dako Cytomation) in a nonmetal-containing plastic-made pressure cooker and irradiated in a microwave oven for 15 minutes (maximum 500 W). After irradiation, sections were rinsed under running water for 2 minutes, soaked in 3% H_2_O_2_ methanol solution for 5 minutes, and then soaked in 5% BSA for 1 minute. After that, M.O.M. mouse Ig blocking reagent was applied and incubated for 1 hour. Primary antibody was diluted to a previously determined optimal concentration in M.O.M. diluent. The diluted antibody was applied to the tissue sections in a moist chamber and irradiated intermittently for 30 minutes (250 W, 4 seconds on, 3 seconds off). After three washes with Tris-buffered saline containing 1% Tween (TBS-T) for 5 minutes, peroxidase-conjugated Envision kit for mouse (Envision-PO, Envision System, Dako Cytomation) was used on the appropriate specimens in the moist chamber. Irradiation was then performed intermittently for 30 minutes as described above. After washing 5 times with TBS, the sections were immersed in DAB solution (Sigma-Aldrich, St. Louis, MO, USA) with H_2_O_2_ and counterstained with hematoxylin (Dako Cytomation) and mounted under coverslips.

Immunopositivity for HIF-1*α* in the tubular cells of the cortex was counted using 18 fields per group, and the average number per field was determined.

### 2.8. Statistical Analysis

All values were presented as mean ± S.D., and were analyzed by one-way analysis of variance (ANOVA) followed by Dunnett's test. *P* < .05 was accepted as statistically significant.

## 3. Results

### 3.1. Body Weight, Kidney Weight, Blood Pressure and Urinary Volume

Table 2 shows the changes in body weight, kidney weight, blood pressure and urinary volume of the rats during the 1-week experimental period. The final body weights of the 5/6Nx groups were significantly lower than that of the sham group. There were no significant differences between baseline and final body weights in the 5/6Nx groups. The remnant kidney weights among the 5/6Nx groups did not change after the 1-week treatment. Blood pressures among all groups also did not change during the 1-week treatment. Urinary volumes of the 5/6Nx groups increased significantly compared to the sham group. There were no significant differences between baseline and final urinary volumes in the 5/6Nx groups.

### 3.2. Serum and Urine Biochemical Parameters

Table 3 shows the effects of hachimijiogan on serum and urine biochemical parameters. BUN levels of the control and low-dose hachimijiogan groups were significantly higher than that of the sham group. There were no significant differences in BUN levels among the four 5/6Nx groups. The s-Cre level of the control group was significantly higher than that of the sham group. There were no significant differences in s-Cre levels among the four 5/6Nx groups. Urinary protein excretion of the control group was significantly increased compared to that of the sham group, and those of the high-dose hachimijiogan and candesartan cilexetil groups were significantly decreased compared to that of the control group. Ccr levels of the four 5/6Nx groups were significantly decreased compared to that of the sham group. The 8-OHdG level of the control group was significantly higher than that of the sham group, and those of the low-dose hachimijiogan, high-dose hachimijiogan, and candesartan cilexetil groups were significantly lower than that of the control group.

### 3.3. Renal Cortical Hypoxia-Related Factors

**Figure 2:**
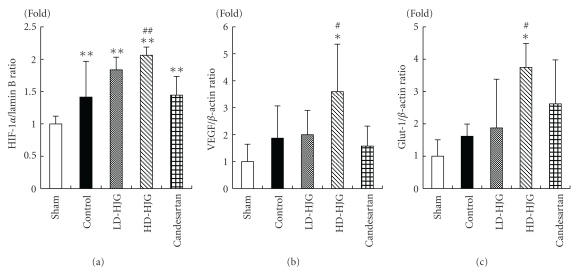
Measurement of renal cortical hypoxia-related factors in remnant kidney treated with hachimijiogan. The volume of HIF-1*α* protein of the four 5/6Nx groups increased significantly compared to that of the sham group, and that of the high-dose hachimijiogan group increased significantly compared to that of the control group (a). The mRNA levels of VEGF and Glut-1 of the high-dose hachimijiogan group increased significantly compared to those of the sham and control groups (b, c). (a) HIF-1*α* protein, (b) VEGF mRNA, (c) Glut-1 mRNA, LD-HJG: low-dose hachimijiogan, HD-HJG: high-dose hachimijiogan. Data represent mean ± S.D. (*n* = 8–10). *P < .05, **P < .01 versus sham group, ^#^ *P* < .05, ^##^ *P* < .01 versus control group.

The volumes of HIF-1*α* protein of the four 5/6Nx groups were significantly increased compared to that of the sham group, and that of the high-dose hachimijiogan group was significantly increased compared to that of the control group (Figure 2(a)). Figures 2(b) and 2(c) show the effects of hachimijiogan on renal mRNA levels of VEGF and glucose transporter-1 (Glut-1). In the high-dose hachimijiogan group, the mRNA levels of VEGF and Glut-1 were significantly increased compared to those of the sham and control groups.

**Figure 3:**
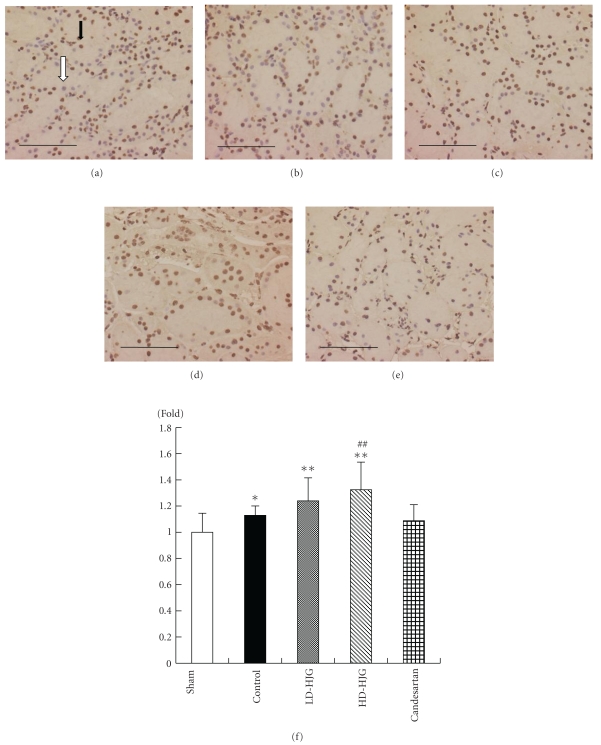
Immunohistochemical studies showed that the expression of HIF-1*α* of the high-dose hachimijiogan group increased compared to that of the control group (a–e). The black arrow indicates an HIF-1*α*-positive cell stained brown, and the white arrow indicates an HIF-1*α*-negative cell stained blue. The number of HIF-1*α*-positive cells in the high-dose hachimijiogan group increased significantly compared to that in the control group. (a) sham, (b) control, (c) low-dose hachimijiogan, (d) high-dose hachimijiogan, (e) candesartan cilexetil. (magnification 400x, scale bar: 100 *μ*m), (f) Comparison with percentage of HIF-1*α*-positive cells/field in the every group. Data represent mean ± S.D. *P < .05, **P < .01 versus sham group, ^##^ *P* < .01 versus control group.

Immunohistochemical studies also showed that the expression of HIF-1*α* of the high-dose hachimijiogan group was increased compared to that of the control group (Figures 3(a)–3(e)). There was a significantly large number of HIF-1*α*-positive cells in the high-dose hachimijiogan group compared to the control group (Figure 3(f)).

## 4. Discussion

Recently, as a final common pathway of various renal diseases, attention has been focused on tubulointerstitial hypoxia. It has been reported that the hypoxia of renal tissue was caused by decreasing peritubular capillary blood flow due to renal fibrosis, abnormal production of vasoactive substance, anemia, and so on [15]. HIF, a heterogeneous nuclear ribonucleoprotein, is an important defense factor against tissue hypoxia. Activation of HIF and numerous downstream target genes protect tissues from hypoxia [6]. Under normoxic conditions, HIF-1*α* subunit is hydroxylated by prolyl hydroxylase (PHD). Hydroxylation is promoted by von Hippel-Lindau tumor suppressor protein binding to HIF-1*α* subunit. As a result, HIF-1*α* subunit is destroyed by proteasome. As PHD activity is decreased under hypoxic condition, HIF-1*α* subunit heterodimerizes with the constitutively expressed HIF-1*β* subunit. The heterodimeric HIF translocates into the nucleus, activating gene transcriptions, such as angiogenesis, cell metabolism, cell growth, apoptosis, and so on [16]. However, it has been reported that activation of HIF becomes less responsive to renal hypoxia in advanced renal dysfunction [17, 18]. It has also been reported that oral administration of cobalt chloride, which activates HIF, ameliorates renal injury in diabetic nephropathy of SHR/NDmer-cp rats [7], and that VEGF plays an important role in capillary repair in damaged glomeruli in glomerulonephritis rats induced by injection of anti-Thy-1.1 antibody [8]. On the basis of these reports, it is suggested that the treatments against hypoxia are useful for suppressing the progression of CKD.

The 5/6 nephrectomized rats we used in this study are a typical model of progressive renal disease. The initial change in this model causes increasing glomerular capillary pressure associated with a relative decrease in the expansion of efferent arterioles, resulting in glomerular hyperfiltration and hypertension. Glomerular hyperfiltration activates the renin-angiotensin system, eventually leading to glomerular sclerosis [19]. However, it has been reported recently that the deterioration of renal function in this model is correlated with an interstitial damage rather than a glomerular damage, and that chronic tissue hypoxia due to the initial reduction of blood flow causes the progression of renal failure [12]. Furthermore, it has been reported that the continuous infusion of dimethyloxalylglycine (DMOG), an activator of HIF, suppressed the increase of proteinuria in this model [20].

There have been some reports regarding the protective effects of hachimijiogan on renal function. Hachimijiogan had antihypertensive and renal-protective effects on Dahl salt-sensitive hypertensive rats, and its mechanism was suspected to enhance the production of prostaglandin E_2_ [21]. Hachimijiogan had protective effects on diabetic nephropathy rats induced by streptozotocin injection, with its mechanism being suspected of improving lipid metabolism, glucose metabolism, and oxidative stress [22]. Hachimijiogan had renal-protective effects by improving oxidative stress and suppressing expression of fibronectin and TGF-*β* in spontaneously diabetic nephropathy rats [10]. Hachimijiogan also had renal-protective effects on 5/6 nephrectomized rats by a mechanism thought to reduce uremic toxins associated with oxidative stress [11]. Morroniside is a main component of Corni Fructus, which is contained in hachimijiogan, and was reported to have a renal-protective effect on diabetic nephropathy rats by inhibiting the production of advanced glycation end product and oxidative stress [23].

In this study, the levels of BUN, s-Cre, and urinary protein excretion of the control group were significantly higher than those of the sham group whereas the level of Ccr of the control group significantly decreased compared to that of the sham group. The HIF-1*α* protein level of the control group increased compared to that of the sham group one week after nephrectomy, as previously reported [12]. 

On the other hand, administration of high-dose hachimijiogan significantly reduced urinary protein excretion and elevated the HIF-1*α* protein level in the renal cortex. Therefore, it was suggested that hachimijiogan had an activating effect on HIF. This was also supported by the observations of increased VEGF mRNA and Glut-1 mRNA. Immunohistological examination of renal tubular epithelial cells also showed an increase in HIF-1*α* by the administration of hachimijiogan.

Administration of candesartan cilexetil, which has renal-protective effects [24, 25], significantly decreased urinary protein excretion. However, the HIF-1*α* protein level and the expressions of VEGF mRNA and Glut 1 mRNA did not increase by candesartan cilexetil administration. It had been reported that HIF activation in renal tissue decreases by the administration of candesartan cilexetil [12]. It is supposed that the main mechanism contributing to the attenuation of proteinuria by the administration of ARB is a reduction in glomerular capillary pressure, as previously reported [26].

It has already been reported that oxidative stress increased in patients with CKD [27]. In the state of uremia, due to the increase of reactive oxygen species in the vascular endothelium, a reduction in glutathione [28] and an increase in releasing reactive oxygen species from leukocytes [29] have been reported. It has also been reported that the activation of the renin-angiotensin system suppresses the expression of super oxide dismutase (SOD) [30]. This oxidative stress has been reported to have the possibility of inhibiting the activation of HIF [31]. Therefore, the involvement of oxidative stress was examined by measuring urinary 8-OHdG. The results showed that, compared with the sham group, the control group had a significantly increased level of urinary 8-OHdG, and the hachimijiogan and candesartan cilexetil groups had significantly decreased levels of urinary 8-OHdG. The antioxidant effects of hachimijiogan have been reported [10, 11]. Although ARB has also been reported to have antioxidant activity [32], the HIF activation in the candesartan cilexetil group did not increase in this study. Therefore, the mechanism of increasing HIF of the hachimijiogan group was considered to be a direct effect of hachimijiogan. 

There are various reports about natural products that have an effect on HIF activation [33]. Concerning phenolic compounds, quercetin, contained in red wine, has been reported to have the effect of HIF activation on cultured murine brain endothelial cells in normoxia [34]. Green tea extract and its major component epigallocatechin gallate (EGCG) have been reported to activate HIF in human prostate cancer cells (PC-3ML) by inhibiting the degradation with PHD [35]. However, EGCG has also been reported to have a suppressive effect on HIF activation in HeLa cells and HepG2 cells by inhibiting the PI3K-AKT-mTOR pathway [36]. It is suggested that the activations of HIF and VEGF depend on the kinds of cultured cells or culture conditions. Moreover, there are as yet few reports on the study of the activation of HIF in vivo.

## 5. Conclusion

**Figure 4:**
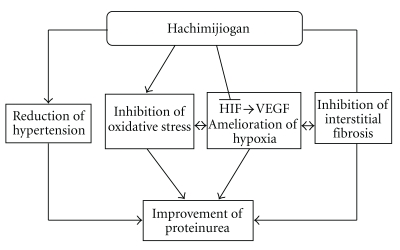
Hypothetical representation of the effects of hachimijiogan on renal dysfunction.

It has been suggested that hachimijiogan has a renal-protective effect through the influence of HIF activation. We summarized our hypothetical representation that explains the effects of hachimijiogan on renal dysfunction in Figure 4. Further experiments are needed to determine the active components and the mechanisms of the activation of HIF, and to study the long-term effects of hachimijiogan in vivo. Meanwhile, in terms of renal treatment via HIF, the hachimijiogan treatment is considered as a new therapeutic tool in addition to the existing renal treatments.

## Figures and Tables

**Table 1:** Sequences for RT-PCR primers.

Genes	Forward	Reverse
*β*-actin	GCCAACCGTGAAAAGATGAC	AGGCATACAGGGACAACACA
VEGF	TTACTGCTGTACCTCCAC	ACAGGACGGCTTGAAGATA [12]
Glut-1	AGGTGTTCGGCTTAGACTC	GAAGGGCAACAGGATACAC

VEGF: vascular endothelial growth factor, Glut-1: glucose transporter 1.

**Table 2:** Physiological data of experimental animals.

Group	Body weight (g)		Kidney weight (g/100g BW)	Systolic blood pressure (mmHg)		Diastolic blood pressure (mmHg)		Urine volume (mL/day)	
	Baseline	Final		Baseline	Final	Baseline	Final	Baseline	Final
Sham	297.1 ± 11.0	325.5 ± 12.0	0.311 ± 0.04	121.4 ± 14.2	113.7 ± 10.8	56.4 ± 13.5	62.6 ± 16.2	5.7 ± 3.1	6.4 ± 2.8
Control	261.7 ± 22.9	291.6 ± 26.4*	0.331 ± 0.05	124.3 ± 5.8	127.7 ± 8.2	69.7 ± 6.7	59.1 ± 12.2	20.3 ± 6.6**	21.1 ± 6.1**
LD-HJG	253.6 ± 17.6	275.9 ± 24.3*	0.341 ± 0.03	132.9 ± 27.1	130.0 ± 17.2	74.2 ± 19.1	62.8 ± 16.2	17.6 ± 6.7**	14.1 ± 6.3**
HD-HJG	257.5 ± 16.6	295.6 ± 16.4*	0.354 ± 0.04	122.8 ± 15.6	126.3 ± 8.9	70.1 ± 17.3	56.9 ± 6.1	20.6 ± 8.3**	18.0 ± 7.1**
Candesartan	264.3 ± 14.6	276.1 ± 16.6*	0.343 ± 0.03	121.4 ± 14.6	119.7 ± 16.2	70.9 ± 15.8	58.5 ± 13.1	20.2 ± 13.4**	16.5 ± 12.8**

LD-HJG: low-dose hachimijiogan; HD-HJG: high-dose hachimijiogan.

Baseline: before drug administration; Final: after 1 week of drug administration.

Data represent mean ± S.D. (* n* = 8–10). *P < .05, **P < .01 versus sham group.

**Table 3:** Effect of hachimijiogan on renal functional parameters.

	s-Alb (mg/dL)	BUN (mg/dL)	s-Cre (mg/dL)	u-Pro (mg/day)	Ccr (mL/min/kg BW)	8-OHdG (ng/day)
Sham	834.8 ± 110.9	15.6 ± 1.9	0.32 ± 0.14	13.01 ± 7.29	5.03 ± 3.07	325.9 ± 106.5
Control	887.1 ± 67.4	50.6 ± 36.7**	1.44 ± 1.32**	35.34 ± 20.62**	1.88 ± 0.94**	840.4 ± 252.4**
LD-HJG	895.8 ± 25.7	42.0 ± 27.1*	0.93 ± 0.59	25.15 ± 15.32	2.13 ± 1.40**	582.9 ± 201.8^#^
HD-HJG	833.9 ± 82.2	39.6 ± 25.7	0.87 ± 0.61	19.73 ± 5.41^#^	2.11 ± 0.86**	550.9 ± 210.5^#^
Candesartan	840.8 ± 92.5	33.8 ± 12.3	0.88 ± 0.49	15.41 ± 5.92^##^	1.65 ± 0.64**	484.2 ± 171.3^##^

LD-HJG: low-dose hachimijiogan; HD-HJG: high-dose hachimijiogan.

Data represent mean ± S.D. (*n* = 8–10).

*P < .05, **P < .01 versus sham group.

^#^ *P* < .05, ^##^ *P* < .01 versus control group.
